# Korean Red Ginseng Plays an Anti-Aging Role by Modulating Expression of Aging-Related Genes and Immune Cell Subsets

**DOI:** 10.3390/molecules25071492

**Published:** 2020-03-25

**Authors:** Kun Kuk Shin, Young-Su Yi, Jin Kyeong Kim, Haeyeop Kim, Mohammad Amjad Hossain, Jong-Hoon Kim, Jae Youl Cho

**Affiliations:** 1 Department of Integrative Biotechnology, Sungkyunkwan University, Suwon 16419, Korea; shuka337@naver.com (K.K.S.); rosekim95@naver.com (J.K.K.); rlagoduq7283@naver.com (H.K.); 2 Department of Life Sciences, Kyonggi University, Suwon 16227, Korea; ysyi@kgu.ac.kr; 3 Department of Veterinary Physiology, College of Medicine, Chonbuk National University, Iksan 54596, Korea; mamjadh2@gmail.com

**Keywords:** korean red ginseng, anti-aging, thymic involution, aging-related genes, immune cell population

## Abstract

Despite previous reports of anti-aging effects of Korean red ginseng (KRG), the underlying mechanisms remain poorly understood. Therefore, this study investigated possible mechanisms of KRG-mediated anti-aging effects in aged mice. KRG significantly inhibited thymic involution in old mice. Interestingly, KRG only increased protein expression, but not mRNA expression, of aging-related genes Lin28a, GDF-11, Sirt1, IL-2, and IL-17 in the thymocytes of old mice. KRG also modulated the population of some types of immune cells in old mice. KRG increased the population of regulatory T cells and interferon-gamma (IFN-γ)-expressing natural killer (NK) cells in the spleen of old mice, but serum levels of regulatory T cell-specific cytokines IL-10 and TGF-β were unaffected. Finally, KRG recovered mRNA expression of Lin28a, GDF-11, and Sirt1 artificially decreased by concanavalin A (Con A) in both thymocytes and splenocytes of old mice without cytotoxicity. These results suggest that KRG exerts anti-aging effects by preventing thymic involution, as well as modulating the expression of aging-related genes and immune cell subsets.

## 1. Introduction

Aging is a biological process characterized by progressive alteration of body tissues, an inability to functionally adapt, and the accumulation of deficits at various organs, leading to a decline in physiological function, age-related diseases, and death [1,2,3]. Aging also involves gradual deterioration of the immune system in the body, known as immunosenescence, which is the result of inflammaging (an imbalance between inflammatory and anti-inflammatory responses) [4,5], oxidative stress [6], remodeling of the immune system [7], apoptosis and upregulation of pro-inflammatory cytokines [7], and differential expression of aging-related genes [8].

*Panax ginseng*, also known as Korean ginseng, is a perennial plant that has long been used as a traditional herbal medicine in the world, especially in far-eastern Asian countries like Korea, China, and Japan [9,10,11]. Since fresh ginseng easily decays at room temperature, ginseng is processed to red ginseng by steaming and drying to a dark red color. Korean red ginseng (KRG) has been demonstrated to have higher pharmacological activities and lower side effects compared to fresh ginseng [12]. Many studies have revealed that KRG has a critical impact on various biological and disease conditions through immune-boosting, antioxidant, neuroprotective, anti-diabetic, hepatoprotective, autophagy-regulatory, and anti-cancer effects [13,14,15,16]. Accumulating evidence indicates that KRG also has anti-aging effects and can extend the life span of organisms [17,18,19,20], but the underlying molecular and cellular mechanisms remain poorly understood. Therefore, this study aimed to investigate KRG-mediated anti-aging effects and the associated underlying molecular and cellular mechanisms in aged mice.

## 2. Results and Discussion

### 2.1. KRG Inhibited Aging-Related Thymic Involution

**Figure 1:**
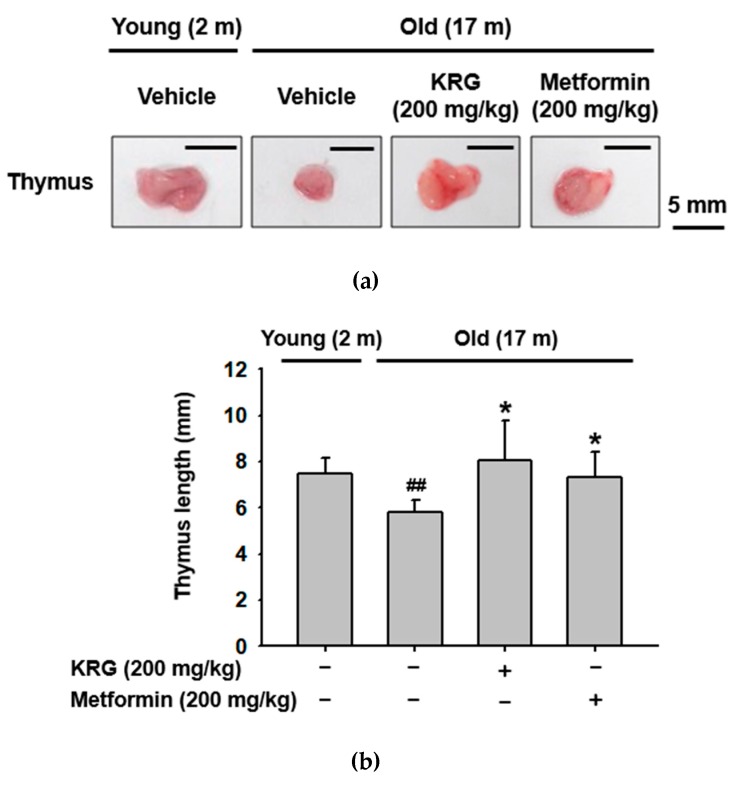
Korean red ginseng (KRG) inhibited aging-related thymic involution. (**a**) Thymi were excised from young (2 months old) and old (17 months old) mice orally dosed with KRG (200 mg/kg) or metformin (200 mg/kg) and photographed. (**b**) Thymic size (length) of the mice (n = 6) was measured and plotted. Scale bar = 5 mm. ^##^*p* < 0.01 compared to vehicle-administered control young mice, and *p < 0.05 compared to vehicle-administered control old mice.

Aging induces the gradual deterioration of the immune system, known as immunosenescence, which results in the alteration of immune functions, including immune deficiency, autoimmunity, and an imbalance of inflammation [21,22,23]. One of the hallmarks of immunosenescence is apoptosis-induced thymic involution, which reduces the T-cell repertoire and leads to accumulation of effector T-cells and autoimmunity [24,25]. Therefore, the effect of KRG on aging-related thymic involution was evaluated in old mice. As expected, thymus size was markedly smaller in old mice (17 months old) compared to young mice (2 months old); however, thymus size in KRG-administered old mice was comparable to that of young mice (Figure 1a). Metformin, a medication for the treatment of type 2 diabetes, has been demonstrated to play an anti-aging role by preventing oxidative stress-induced DNA damage and inflammation, which improves aging outcomes [26,27]. Similar to KRG, thymic size in metformin-treated old mice was as big as that in young mice (Figure 1a). Overall, KRG and metformin significantly inhibited the decrease in thymic length seen in untreated old mice (Figure 1b). These results suggest that KRG plays an anti-aging role by inhibiting aging-related thymic involution in mice.

### 2.2. KRG Altered Protein Expression, But Not mRNA Expression of Aging-Related Genes in Aged Mice

**Figure 2:**
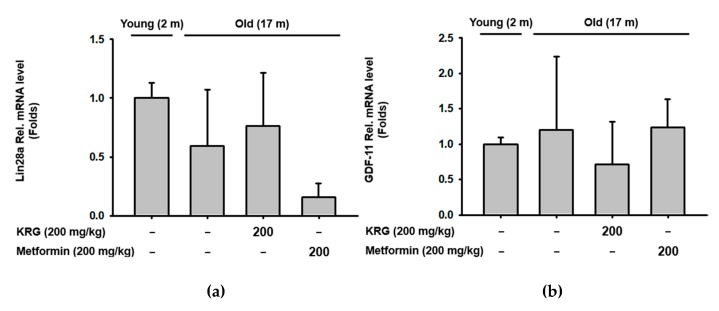

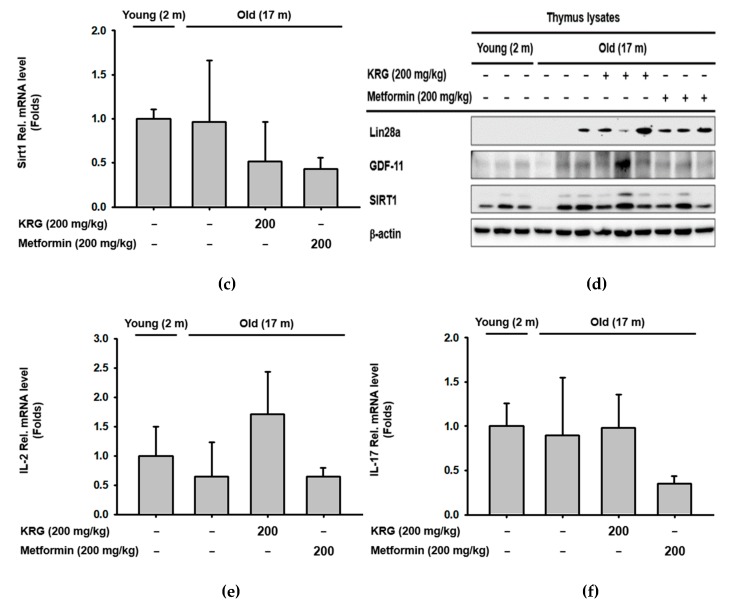
KRG did not alter the expression of aging-related genes in aged mice. (**a–c**) mRNA expression of Lin28a, GDF-11, and Sirt1 in the thymocytes of young and old mice dosed with KRG (200 mg/kg) or metformin (200 mg/kg) was determined using quantitative real-time PCR. (**d**) Protein expression of Lin28a, GDF-11, and Sirt1 in the thymocytes of young and old mice dosed with KRG (200 mg/kg) or metformin (200 mg/kg) was determined using Western blot analysis. (**e**,**f**) mRNA expression of IL-2 and IL-17 in the thymocytes of young and old mice dosed with KRG (200 mg/kg) or metformin (200 mg/kg) was determined using quantitative real-time PCR.

The anti-aging effect of KRG was next investigated by evaluating the expression of aging-related genes in old mice. Lin28a, an RNA-binding protein that is highly expressed in embryonic stem cells, helps generate energy for cellular functions through glycolytic metabolism, and a decrease in its expression is a hallmark of the aging process [28]. Growth Differentiation Factor-11 (GDF-11), a member of the transforming growth factor family, has been reported as a rejuvenation factor that reverses age-related decline of tissue functions [29,30,31,32] and is highly expressed in young animals [33]. Sirtuin 1 (SIRT1), an NAD-dependent deacetylase, helps prevent age-related DNA damage and telomere shortening by inducing telomerase reverse transcriptase activity [34,35]. Knockdown of its expression in young cells induces cellular senescence and proliferation, whereas, its overexpression in aged cells reverses senescence phenotypes [36]. Taken together, these findings led to an investigation into the effect of KRG on the expression of aging-related genes in the thymus of old mice by quantitative real-time PCR analysis. mRNA expression levels of these genes in thymocytes were neither different between young and old mice nor statistically changed by KRG in the thymocytes of old mice (Figure 2a–c). Interestingly, unlike mRNA expression, KRG induced protein expression of Lin28a, GDF-11, and Sirt1 in the thymocytes of old mice (Figure 2d). The reason why KRG induced protein expression of these genes only and not mRNA expression is not clear, and requires further investigation.

Interleukin (IL)-2 is a key molecule produced by helper T cells that induces the differentiation and function of many types of immune cells [37,38]. In the thymus, IL-2 promotes the differentiation of regulatory T cells to prevent autoimmunity [39,40,41,42]. IL-17, a cytokine also produced by helper T cells, plays a role in promoting host defense, pro-inflammatory, and allergic responses [43]. Studies have reported that the expression of IL-2 decreases while that of IL-17 increases during the aging process [44,45,46]. Given this evidence, the effect of KRG on the expression of these cytokines in the thymus of old mice was investigated by quantitative real time PCR analysis. KRG did not affect mRNA expression of either IL-2 or IL-17 in the thymocytes of old mice (Figure 2e,f).

In summary, these results suggest that although KRG plays an anti-aging role by preventing thymic involution (Figure 1), KRG only increases protein expression, but not mRNA expression, of aging-related genes such as Lin28a, GDF-11, and Sirt1, as well as aging-related cytokines such as IL-2 and IL-17 in the thymus of old mice. These results raise the necessity of investigating how KRG inhibits thymic involution through mechanisms other than altering the expression of aging-related genes and cytokines in the aged thymus. Moreover, further research on the effects of KRG on the expression of these aging-related genes in other types of immune organs and cells that play critical roles in the aging process is needed.

### 2.3. KRG Regulated the Population of Thymic and Splenic Immune Cells in Aged Mice

Previous studies demonstrated that immune system impairment with the aging process is associated with alterations in the quantity of various immune cell subsets, such as CD4 T cells, CD8 T cells, and natural killer (NK) cells [47,48,49,50]. Accordingly, the effect of KRG on alterations to the quantity of various immune cell subsets was next investigated by flow cytometry.

**Figure 3:**
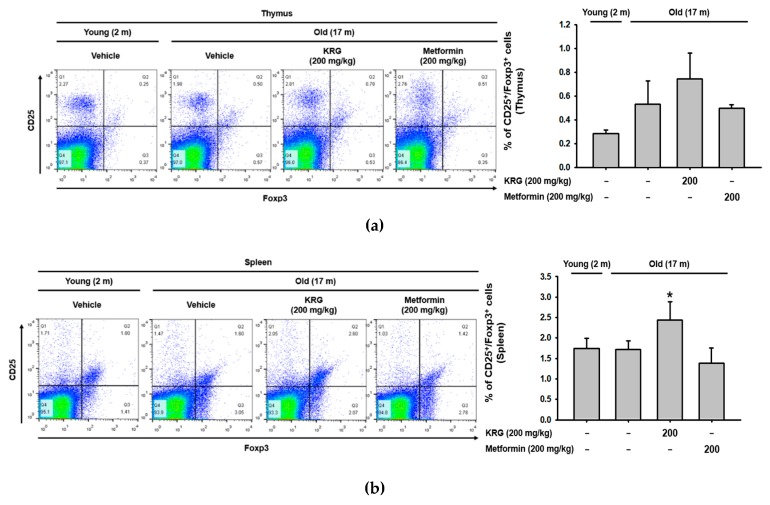

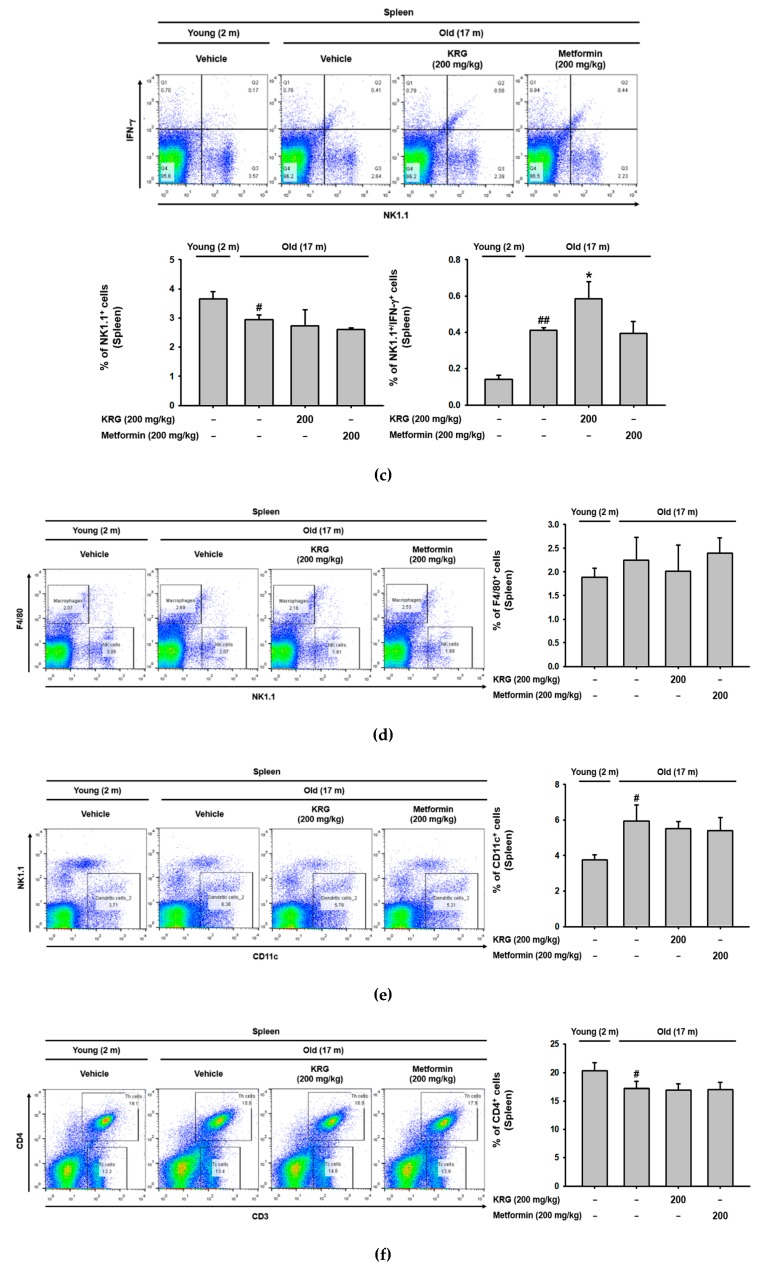
KRG regulated the population of thymic and splenic immune cells in aged mice. (**a**) Total thymic cells were stained for CD25 and Foxp3, and CD25^+^/Foxp3^+^ cells were analyzed using flow cytometry and plotted. (**b**) Total splenic cells were stained for CD25 and Foxp3, and CD25^+^/Foxp3^+^ cells were analyzed using flow cytometry and plotted. (**c**) Total splenic cells were stained for NK1.1 and IFN-γ, and NK1.1^+^/IFN-γ^+^ cells were analyzed using flow cytometry and plotted. (**d**) Total splenic cells were stained for F4/80 and NK1.1, and F4/80^+^ cells were analyzed using flow cytometry and plotted. (**e**) Total splenic cells were stained for CD11c and NK1/1, and CD11c^+^ cells were analyzed using flow cytometry and plotted. (**f**) Total splenic cells were stained for CD4 and CD3, and CD4^+^ cells were analyzed using flow cytometry and plotted. ^#^*p* < 0.05, ^##^*p* < 0.01 compared to vehicle-administered control young mice, and *p < 0.05 compared to vehicle-administered control old mice.

Regulatory T cells are suppressive T cells with an immunosuppressive function that helps maintain immune tolerance to self-antigens and prevents autoimmunity. The effect of aging on the pool of regulatory T cells is still poorly understood, hence, the effect of KRG on the numbers of regulatory T cells was first evaluated in the primary lymphoid organs of the thymus and spleen of old mice. The number of CD25^+^/Foxp3^+^ regulatory T cells in both the thymus (Figure 3a) and spleen (Figure 3b) was not statistically different between young and old mice. However, KRG significantly increased the quantity of splenic regulatory T cells (Figure 3b), but not thymic regulatory T cells, in old mice (Figure 3a). These results might indicate that KRG inhibits signs of immunosenescence, such as autoimmunity and inflammaging, by increasing the number of regulatory T cells in the spleen where T cells are activated, but not in the thymus where T cells mature and differentiate. KRG may increase the population of regulatory T cells by promoting their function rather than their differentiation.

NK cells are a type of cytotoxic immune cell that plays a critical role in the elimination of pathogen-infected cells, and the effect of KRG on the NK cell population was next evaluated in the spleen of old mice. Total numbers of splenic NK1.1^+^ NK cells were reduced in old mice compared to young mice, but were not changed by KRG in old mice (Figure 3c). We further examined the effect of KRG on the population of functional NK cells in the spleen of old mice. The population of functional splenic NK cells expressing interferon-gamma (IFN-γ; NK1.1^+/^IFN-γ^+^) was increased in old mice compared to young mice and also increased by KRG in old mice (Figure 3c). These results indicate that KRG promotes immunity in old mice by inducing NK cell function rather than increasing the NK cell population.

Macrophages are innate immune cells that eliminate pathogens by phagocytosis, and the effect of KRG on the macrophage population was evaluated in the spleen of old mice. No difference in the population of splenic F4/80^+^ macrophages was observed between young and old mice, and KRG did not alter the population of splenic macrophages in old mice (Figure 3d).

Dendritic cells are antigen-presenting cells that link innate and adaptive immunity, and the effect of KRG on the population of dendritic cells was further evaluated in the spleen of old mice. The population of splenic CD11c^+^ dendritic cells was increased in old mice compared to young mice, but was not altered by KRG in old mice (Figure 3e).

CD4 T cells, also known as helper T cells, are adaptive immune cells that activate other types of immune cells by releasing various cytokines, and the effect of KRG on the population of CD4 T cells was evaluated in the spleen of old mice. The population of splenic CD4^+^ T cells was slightly smaller in old mice compared to young mice, but was not altered by KRG in old mice (Figure 3f).

Taken together, these results suggest that KRG reduces the risks of immunosenescence, such as autoimmunity and inflammaging, by increasing the population of splenic regulatory T cells and promotes immunity by inducing the function of splenic NK cells through increasing the population of the functionally active IFN-γ-expressing splenic NK cells in old mice. Further studies investigating the molecular mechanisms by which KRG regulates the populations of these immune cells are warranted.

### 2.4. KRG Did Not Alter the Production of Regulatory T Cell-Specific Cytokines IL-10 and TGF-β in Aged Mice

**Figure 4:**
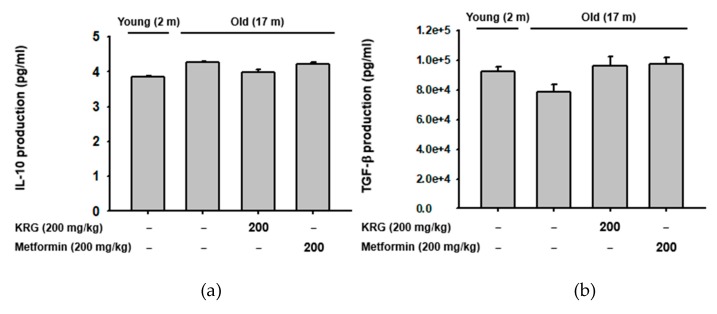
KRG did not alter the production of regulatory T cell-specific cytokines IL-10 and TGF-β in aged mice. Serum level of (**a**) IL-10 and (**b**) TGF-β in young and old mice dosed with KRG (200 mg/kg) or metformin (200 mg/kg) was determined using ELISA.

Since KRG increased the population of splenic CD25^+^/Foxp3^+^ regulatory T cells in old mice (Figure 3b), the effect of KRG on the production of regulatory T cell-specific cytokines, IL-10 and tumor growth factor-β (TGF-β) [51,52,53] was next investigated in old mice. Serum levels of IL-10 and TGF-β were not significantly different between young and old mice (Figure 4a,b). Interestingly, although KRG increased the population of splenic regulatory T cells in old mice (Figure 3b), it did not significantly induce the production of IL-10 and TGF-β (Figure 4a,b). The exact reason for this observation is unclear. However, it may be that KRG only increases the population of splenic regulatory T cells, but does not stimulate the production of regulatory T cell-specific cytokines under normal conditions. Under conditions that activate regulatory T cells, however, KRG might modulate not only the population of regulatory T cells, but also the production of regulatory T cell-specific cytokines IL-10 and TGF-β. This possibility needs further investigation.

### 2.5. KRG Increased the Expression of Aging-Related Genes in Con A-Stimulated T Cells in Aged Mice

**Figure 5:**
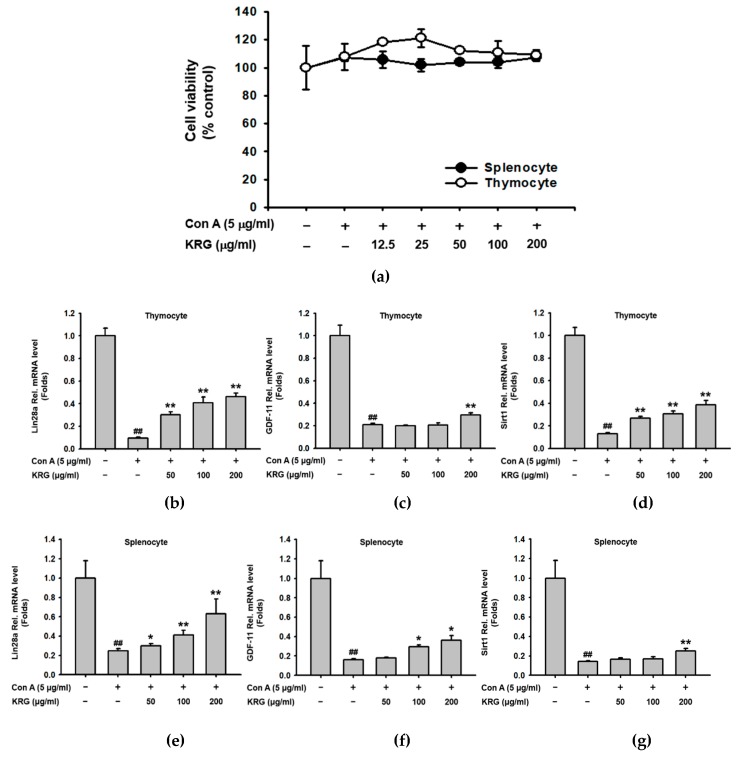
KRG increased the expression of aging-related genes in Con A-stimulated T cells in aged mice. (**a**) Total thymocytes and splenocytes pretreated with Con A (5 μg/mL) for 30 min were treated with the indicated doses of KRG (0–200 μg/mL) for 24 h, and cell viability was determined using a conventional MTT assay. (**b–d**) mRNA expression of Lin28a, GDF-11, and Sirt1 genes in the thymocytes of old mice treated with Con A (5 μg/mL) and KRG (0–200 μg/mL) for 6 h was determined using quantitative real-time PCR. (**e–g**) mRNA expression of Lin28a, GDF-11, and Sirt1 genes in the splenocytes of old mice treated with Con A (5 μg/mL) and KRG (0–200 μg/mL) for 6 h was determined using quantitative real-time PCR. ^##^*p* < 0.01 compared to vehicle-treated control cells, and *p < 0.05, **p < 0.01 compared to Con A-treated control cells.

Aging is strongly associated with the decline of functionally active T cells and the accumulation of T cells hyporesponsive to the effects of activators and mitogens such as concanavalin A (Con A) [54,55]. Therefore, the effect of KRG on the expression of aging-related genes in Con A-stimulated thymocytes and splenocytes of old mice was investigated by quantitative real-time PCR analysis. First, the cytotoxicity of KRG on Con A-stimulated thymocytes and splenocytes of old mice was evaluated, and KRG showed no cytotoxicity on these cells at the doses tested in this study (12.5–200 μg/mL) (Figure 5a). The effect of KRG on the expression of aging-related genes Lin28a, GDF-11, and Sirt1 in Con A-stimulated thymocytes of old mice was next evaluated. KRG dose-dependently increased mRNA expression of Lin28a (Figure 5b), GDF-11 (Figure 5c), and Sirt1 (Figure 5d) that was downregulated by Con A in the thymocytes of old mice. Similar results were observed in splenocytes. KRG dose-dependently increased mRNA expression of Lin28a (Figure 5e), GDF-11 (Figure 5f), and Sirt1 (Figure 5g) that was downregulated by Con A in the splenocytes of old mice. These results are consistent with findings that KRG increased protein expression of these genes in the thymocytes of old mice (Figure 2d), but different than observations that KRG did not increase mRNA expression of these genes in non-stimulated thymocytes of old mice (Figure 2a–c). Why the effect of KRG on mRNA expression of these genes differed between non-stimulated and mitogen-stimulated thymocytes of old mice is unclear, and further study is needed to clarify.

All together, these results indicate that KRG, without cytotoxicity, can inhibit the decline of functionally active T cells and the accumulation of hyporesponsive T cells with age in the thymi and spleens of old mice by increasing the expression of aging-related genes that are expected to be downregulated during the aging process.

## 3. Materials and Methods 

### 3.1. Materials

Korean red ginseng (KRG) was purchased from Korea Ginseng Corp. (Daejeon, Korea), and the information on the composition of KRG was described in Table 1. C57BL/6J young mice (male, 2 months old) and old mice (male, 17 months old) were purchased from Dae Han Bio Link Co., Ltd. (Osong, Korea). Dulbecco’s modified Eagle’s medium (DMEM), phosphate-buffered saline (PBS), streptomycin, penicillin, l-glutamate, and MuLV reverse transcriptase (RT) were purchased from Thermo Fisher Scientific (Waltham, MA, USA). Metformin, 3-(4,5-dimethylthiazol-2-yl)-2,5-diphenyltetrazolium bromide (MTT), Con A, bovine serum albumin (BSA), and sodium azide were purchased from Sigma-Aldrich (St. Louis, MO, USA). TRI reagent^®^ was purchased from Molecular Research Center Inc. (Cincinnati, OH, USA). Primers used for quantitative real-time polymerase chain reaction (PCR) were designed and synthesized at Bioneer Inc. (Daejeon, Korea). Antibodies used for Western blot and flow cytometry analyses were purchased from Cell Signaling Technology (Beverly, MA, USA) and Santa Cruz Biotechnology (Santa Cruz, CA, USA). Enhanced chemiluminescence (ECL) reagent was purchased from AbFrontier Co., Ltd. (Seoul, Korea). IL-10 and TGF-β enzyme-linked immunosorbent assay (ELISA) kits were purchased from R&D Systems, Inc. (Minneapolis, MN, USA).

### 3.2. Animal Husbandry and Experiments

C57BL/6J mice were caged with a 12-h light and dark cycle and fed a pelleted diet and tap water *ad libitum*. For animal experiments, mice (n = 7/group) were orally administered with either vehicle, KRG (200 mg/kg), or metformin (200 mg/kg) once a day for 30 days. The doses of KRG and metformin used in this study were determined as optimal doses based on the previous studies [56,57,58,59,60]. The mice were anaesthetized prior to tissue excision and blood collection by the intraperitoneal injection with ketamine (100 mg/kg) and xylazine (10 mg/kg) according to the previous study [61]. All animal studies were conducted according to the Institutional Animal Care and Use Committee of Sungkyunkawn University (Approved number: 2018-10-16-1). Thymi from each group of mice orally dosed with either KGR (200 mg/kg) or metformin (200 mg/kg) were excised and photographed to measure their size.

### 3.3. Quantitative Real Time PCR

Mice were orally dosed with either KGR (200 mg/kg) or metformin (200 mg/kg). Total thymocytes and splenocytes isolated from the old mice were treated with Con A (5 μg/mL) and the indicated doses of KGR (0–200 μg/mL) for 6 h. Total RNA was isolated from total thymocytes and splenocytes using TRI reagent^®^ according to the manufacturer’s instructions. cDNA was synthesized from total RNA (1 μg) using MuLV RT according to the manufacturer’s instructions and used for quantitative real-time PCR to measure the expression levels of target genes. All primer sequences used for quantitative real-time PCR are listed in Table 2.

### 3.4. Western Blot Analysis

Mice were orally dosed with either KGR (200 mg/kg) or metformin (200 mg/kg). Total thymocytes and splenocytes isolated from old mice were treated with Con A (5 μg/mL) and the indicated doses of KGR (0–200 μg/mL) 24 h. Total thymocyte and splenocyte lysates were prepared by homogenization in cold lysis buffer (20 mM Tris HCl, pH 7.4, 2 mM EDTA, 2 mM ethyleneglycotetraacetic acid, 50 mM β-glycerophosphate, 1 mM sodium orthovanadate, 1 mM dithiothreitol, 1% Triton X-100, 10% glycerol, 10 mg/mL aprotinin, 10 mg/mL pepstatin, 1 mM benzimide, and 2 mM PMSF) at 4 °C for 30 min. Whole thymocyte and splenocyte lysates were subjected to Western blot analysis, and protein targets were detected using the antibodies specific for each target, as previously described [62].

### 3.5. Flow Cytometry Analysis

Mice were orally dosed with either KGR (200 mg/kg) or metformin (200 mg/kg). Total thymocytes and splenocytes from these mice were prepared and washed three times with cold PBS, followed by suspension in flow buffer (1% BSA, 0.1% sodium azide). The cell suspension was incubated with the indicated antibodies on ice for 30 min in the dark, and the fluorescence was detected using a CytoFLEX Flow Cytometer (Beckman Coulter Life Sciences, Indianapolis, IN, USA).

### 3.6. ELISA

Sera of each group of mice orally dosed with either KGR (200 mg/kg) or metformin (200 mg/kg) were collected from whole blood by centrifugation for 1 min. The sera were used for ELISA to measure the amounts of IL-10 and TGF-β in blood according to the manufacturer’s instructions.

### 3.7. Cell Viability Assay

Total thymocytes and splenocytes were treated with Con A (5 μg/mL) and the indicated doses of KGR (0–200 μg/mL) for 24 h, and cell viability was determined by conventional MTT assay, as previously described [63].

### 3.8. Statistical Analysis

In this study, all data are presented as means ± standard error of the mean (S.E.M.) obtained from three independent experiments. Analysis of variance (ANOVA) with Scheffe’s post hoc test or Kruskal-Wallis/Mann-Whitney tests were used to compare data and to assess the significance of group differences. All statistical analyses were conducted using an SPSS program (SPSS Inc., Chicago, IL, USA), and a *p* value less than 0.05 indicated statistical significance.

## 4. Conclusions

Despite a number of studies investigating various pharmacological effects of KRG, only a small number of studies have investigated the anti-aging effect of KRG, and the underlying mechanism has not been clearly demonstrated yet. Therefore, this study aimed to investigate the KRG-mediated in vivo and ex vivo anti-aging effects and to unveil the underlying molecular and cellular mechanisms using the aged mice. KRG showed an in vivo anti-aging effect by suppressing the age-related thymic involution without the significant cytotoxicity, and this KRG-mediated anti-aging effect was achieved by increasing the expression of Lin28a, GDF-11, and SIRT1, as well as the population of the splenic regulatory T cells and IFN-γ-expressing NK cells in the aged mice. In conclusion, these results suggest that KRG plays an anti-aging role by modulating the expression of the age-related genes and the population of the immune cell subsets in the aged mice.

## Figures and Tables

**Table 1:** Information of the composition of KRG (g).

Ingredient	Amount
Ginsenoside Rg1	5.5 mg
Ginsenoside Rb1	
Ginsenoside Rg3	
Carbohydrate	0.33 g

**Table 2:** Primer sequences used for quantitative real-time PCR in this study.

Targets		Sequences (5′→3′)
Lin28a	For	TCGGTGTCCAACCAGCAGTT
	Rev	GGCGGTCATAGACAGGAAGC
GDF-11	For	GATCCTGGACCTACACGACTTC
	Rev	GGCCTTCAGTACCTTTGTGAAC
Sirt1	For	CAGTGTCATGGTTCCTTTGC
	Rev	CACCGAGGAACTACCTGAT
IL-2	For	TTAGGACAGCACAAAGTAAGCG
	Rev	TGAGCTGATGTTAGCTCCCTG
IL-17	For	GCTGACCCCTAAGAAACCCC
	Rev	GAAGCAGTTTGGGACCCCTT
GAPDH	For	CAATGAATACGGCTACAGCAAC
	Rev	AGGGAGATGCTCAGTGTTGG
